# Malaria resistance genes are associated with the levels of IgG subclasses directed against *Plasmodium falciparum* blood-stage antigens in Burkina Faso

**DOI:** 10.1186/1475-2875-11-308

**Published:** 2012-09-04

**Authors:** Sarwat Afridi, Alexandre Atkinson, Séverine Garnier, Francis Fumoux, Pascal Rihet

**Affiliations:** 1 UMR1090 TAGC, INSERM, Marseille, F-13288, France; 2 Aix-Marseille University, Marseille, F-13288, France; 3 UMR-MD3, Marseille, F-13385, France

**Keywords:** HBB, Immune genes, Plasmodium falciparum, Malaria, IgG subclass, Family-based association

## Abstract

**Background:**

*HBB, IL4, IL12*, *TNF, LTA, NCR3* and *FCGR2A* polymorphisms have been associated with malaria resistance in humans, whereas cytophilic immunoglobulin G (IgG) antibodies are thought to play a critical role in immune protection against asexual blood stages of the parasite. Furthermore, *HBB, IL4, TNF,* and *FCGR2A* have been associated with both malaria resistance and IgG levels. This suggests that some malaria resistance genes influence the levels of IgG subclass antibodies.

**Methods:**

In this study, the effect of *HBB, IL4, IL12*, *TNF, LTA, NCR3* and *FCGR2A* polymorphisms on the levels of IgG responses against *Plasmodium falciparum* blood-stage extract was investigated in 220 individuals living in Burkina Faso. The Pearson’s correlation coefficient among IgG subclasses was determined. A family-based approach was used to assess the association of polymorphisms with anti-*P. falciparum* IgG, IgG1, IgG2, IgG3 and IgG4 levels.

**Results:**

After applying a multiple test correction, several polymorphisms were associated with IgG subclass or IgG levels. There was an association of i) haemoglobin C with IgG levels; ii) the FcγRIIa H/R131 with IgG2 and IgG3 levels; iii) *TNF*-863 with IgG3 levels; iv) *TNF*-857 with IgG levels; and, v) *TNF*1304 with IgG3, IgG4, and IgG levels.

**Conclusion:**

Taken together, the results support the hypothesis that some polymorphisms affect malaria resistance through their effect on the acquired immune response, and pave the way towards further comprehension of genetic control of an individual’s humoral response against malaria.

## Background

*Plasmodium falciparum* malaria is a major cause of worldwide mortality and morbidity. Host genetic factors have been shown to influence malaria infection intensity and clinical malaria. Several candidate genes have been associated with resistance against severe malaria, whereas linkage or association analyses mapped several loci controlling mild malaria and/or parasitemia [1]. Noticeably, chromosomes 5q31-q33 and 6p21-p23 have been linked to parasitaemia or mild malaria [2,3], whereas genes located within those chromosomal regions have been associated with parasitaemia, mild or severe malaria [4-10]*.* The *HBB* locus has been shown to be a major locus based on a genome association study for severe malaria, whereas haemoglobin S (HbS) and haemoglobin C (HbC) have been associated with protection against mild and severe malaria in a large number of studies. It should be stressed that a limited number of genes have been associated with mild or severe malaria in several independent studies; these include *FCGR2A* that encodes the human IgG receptor FcγRIIa.

Anti-*P. falciparum* IgG antibodies are thought to play a critical role in immune protection against asexual blood stages of the parasite. Passive transfer of IgG has provided protection against the *P. falciparum* blood stages in humans. *In vitro,* human IgGs that recognize either infected erythrocytes or merozoites act in cooperation with monocytes to eliminate the parasite [11]. Cytophilic IgGs that activate effector cells are, therefore, considered protective, while non-cytophilic IgGs against the same epitope may block the protective effect of the cytophilic ones. This hypothesis has been supported by several immune-epidemiological studies. High levels of the cytophilic IgG3 subclass have been associated with reduced parasitaemia, and protection against mild and severe malaria [12,13]. Interestingly, high levels of IgG2 could be correlated with protection in individuals carrying the H131 variant of monocytes FcγRIIa receptor, which efficiently binds to IgG2. In contrast, high levels of non-cytophilic IgG4 antibodies have been associated with susceptibility to malaria [14]. In this context, several investigators have provided evidence of the genetic control of IgG levels. Twin studies have shown a better concordance in monozygotic twins than in dizygotic twins for IgG levels [15]. In addition, high sib-sib correlations for IgG subclass levels have been detected [16-18]. Further evidence of a genetic component has been provided by a survey conducted in several sympatric ethnic groups having different genetic backgrounds [19]. Some candidate genes have been associated with IgG or IgG subclass levels. These include *HBB, IL4, TNF,* and *FCGR2A,* which have also been associated with both malaria resistance and IgG levels [1,20-25]. This suggests that those genes control the production of cytophilic IgG subclasses. More generally, genes that have been associated with malaria resistance may be associated with the level of protective IgG subclasses. The linkage or association of *HBB, IL4, IL12*, *TNF, LTA,* and *NCR3* with mild malaria or parasitaemia has been previously reported in a population living in Burkina Faso [2,3,6,9,10,26]. The objective of this study was to determine the influence of *HBB, IL4, IL12*, *TNF, LTA, NCR3* and *FCGR2A* polymorphisms on the IgG subclass patterns of antibodies against *P. falciparum* antigens in the same population by using a family-based approach.

## Methods

### Subjects, clinical diagnosis, and parasitological data

The study population consisted of 220 individuals belonging to 34 families living in urban district of Bobo Dioulasso in Burkina Faso, in which infected mosquitoes were detected only during August, September and October; the numbers of infective bites per person and per year was 30. Blood samples were taken from individuals in July at the end of the dry season (P1) and in December at the end of rainy season (P2). The mean age of sibs was 12.1 + 6.2 years (range one to 34 years). The study population and the area of parasite exposure have been described elsewhere [14,27]. Phenotypes and DNA were available for all the individuals. The Medical Authority of Burkina Faso approved the study protocol. Informed consent for multiple immunoparasitological and clinical surveys was obtained individually from all participants.

Febrile episodes were extensively recorded by active case detection during 24 months. For patients with fever, a thick blood film was prepared by the standard procedures. Diagnosis of mild malaria attack was based on *P. falciparum* parasitaemia, fever (axillary temperature more than 37.5°C) and clinical symptoms (headache, aching, vomiting or diarrhoea in the children); in that case no threshold of parasitaemia was used. In the absence of classical symptoms of malaria, and once others pathologies could not be eliminated, only children (age < 15 years) with more than 5,000 parasites per ml and older subjects with more than 2,000 parasites per ml were considered as having had a malaria attack. Each episode of illness was treated according to the recommendation of the CNRFP (Centre National de Recherche et Formation sur le Paludisme) of Burkina Faso. Parasitaemia was checked at the end of the treatment. Subjects who presented at least one mild malaria attack during the survey were considered in the analysis affected, while the others were considered unaffected.

Determination of parasitaemia was described in a previous study [26]. Briefly, each family was visited 20 times during the 24 months of the study, and parasitaemia was measured. In addition, parasitaemia was measured during febrile episodes. The mean number of parasitaemia measurements per subject was 15.2 + 5.1 (range 1–24). Fingerprint peripheral blood samples were taken from all family members present and thick and thin blood films were stained with Giemsa. The parasite determination and numeration were established blindly from two independent readings. Only *P. falciparum* asexual forms were retained to determine parasitaemia. Parasitaemia was defined as the number of parasitized erythrocytes observed per μl in thin blood films.

Maximum parasitaemia was based on a logarithmic transformation of the highest parasitaemia that was measured in each individual during the survey [26]. Mean of adjusted asymptomatic parasitaemia was a logarithmic transformation of the parasitaemia adjusted for seasonal transmission [26], after excluding parasitaemia during febrile episodes. To take into account the seasonality of the transmission, the influence of the date of the visits on ln(1 + parasitaemia) (LP) was evaluated by one way analysis of variance. The mean LP observed during each visit was calculated. The individual LP was then corrected for the visit effect by subtracting from each individual LP the mean LP of the corresponding visit.

Since age influenced mild malaria attack, maximum parasitaemia, and the mean of adjusted parasitaemia, the age effect was further taken into account. Linear regression was carried out to calculate the residual for maximum parasitaemia and the mean of adjusted parasitaemia, whereas logistic regression was used to calculate the residual for mild malaria. The residuals were used as phenotypes for linkage and association analyses.

### IgG sub-class phenotype determination

The measurements of IgG subclasses directed against *P. falciparum* blood-stage extracts were previously performed; the data sets previously reported was used [14]. Briefly, the *P. falciparum* W2 strain (Southeast Asia) was maintained and synchronized. Schizonts were isolated from infected red blood cells based on a treatment with saponin, were sonicated on ice in phosphate-buffered saline containing protease inhibitors. Sonicates were centrifuged, and the supernatants were filtered through a 0.22 μm-pore-size membrane, and were aliquoted and stored at −70°C until use. Enzyme-linked immunosorbent assay (ELISA) plates (Nunc) were coated with 1 μg of *P. falciparum* extract/ml in sodium carbonate buffer (100 mM, pH 9.6). Plates were saturated with 3% bovine serum albumin in phosphate-buffered saline. Serum dilutions were incubated for 16 h at 4°C (1:20 for IgG2 and IgG4, 1:100 for IgG1 and IgG3, and 1:400 for IgG). The following monoclonal antibodies were used: anti-IgG1 (clone 8c/6-39; The Binding Site), IgG2 and IgG3 (clone HP 6002 and HP 6050; Clinisciences), and IgG4 (clone RJ4; Immunotech). Total IgG was detected using a goat F(ab′)_2_ anti-human IgG (Jackson Laboratories). The anti-IgG1 and -IgG were conjugated to alkaline phosphatase, the anti-IgG2 and -IgG3 were biotinylated, and the anti-IgG4 was unlabelled. F(ab′)_2_ anti-mouse IgG conjugated to alkaline phosphatase was used for IgG4 detection. Signal amplification was performed for IgG2 and IgG3 detection by using streptavidin and biotinylated alkaline phosphatase (Pierce); the sensitivity of the assay was 30-fold higher than that of the assay using the same monoclonal antibodies conjugated to alkaline phosphatase. After 2 h of incubation at room temperature, enzymatic activities were revealed by *p*-nitrophenyl phosphate (Sigma) (1 mg/ml) in Tris buffer (pH 9.6). The optical densities were read at 405 nm using a DIAS automatic plate reader (Dynex Technology). A pool of 200 sera equally diluted was used to draw a standard curve; the same serum pool was used for IgG, IgG1, IgG2, IgG3 and IgG4 measurements. Fifty samples that were randomly selected were titrated; the titration curves were parallel to the standard curve for each IgG subclass. All tests were done in duplicate, and antibody levels were calculated by using the standard curve and were expressed as arbitrary units (AU). To allow for zero values in further analyses, a logarithmic transformation was applied based on log (1 + AU) (LAU) to the AU. The mean LAU and standard deviation were calculated at P1 and at P2. To correct the individual LAU for the visit effect, the LAU was standardized at P1 and at P2, and the mean of adjusted LAU (MALAU) was calculated for each subject, as previously described. The influence of age and sex on MALAU was further evaluated using polynomial regression; the age was considered as a continuous variable. There was an effect of age on IgG subclass and IgG levels (P < 0.01), whereas there was no influence of sex. Age was retained for adjustment for each IgG subclass. The residual was the phenotype used in the statistical analyses. IgG, IgG1, IgG2, IgG3 and IgG4 phenotypes were calculated for 193 individuals.

### Genotyping

Blood samples were taken by venipuncture. The haemoglobin genotypes were identified by electrophoresis of red blood cell lysates on acetate membrane at an alkaline pH. Acetate sheets were stained with Ponceau red. This yielded discrimination of haemoglobin A (HbA), S (HbS) and C (HbC).

DNA was extracted from mononuclear cells separated by the Ficoll–Hypaque density gradient as described [27]. Samples were first subject to prior whole-genome amplification by primer extension pre-amplification [28]. For *IL4*, *FCGR2A*, *NCR3*, *TNF*, *LTA*, and *IL12B* polymorphisms, the data sets previously reported were used [6,9,10,29,30]. Briefly, genotypes for *NCR3*, *TNF*, *LTA*, and *IL12B* polymorphisms have been obtained by sequencing with a CEQ 8000 automated fluorescent sequencer (Beckman Coulter, Roissy CDG, France), whereas *FCGR2A* H/R131 genotypes was determined by using PCR and allele-specific restriction enzyme digestion methods as previously described [31]. The call rate was higher than 88% for all the polymorphisms, based on the 193 individuals, for which the IgG subclass levels have been measured; the median call rate was 94%.

### Statistical analyses

The correlation among IgG subclasses was evaluated using Pearson's correlation coefficient. In addition, combined association and linkage analyses were carried out using the family-based association test (FBAT) approaches [32]. The FBAT statistics, which use data from sibships in nuclear families, takes into account sibling correlations. The default null hypothesis is no linkage or no association and the statistics under this hypothesis calculates the distribution of offspring genotypes that are conditional on parental genotypes and on trait values. FBAT calculates a Z score and a two-side P-value based on normal distribution. Multiple test corrections was further performed using the false discovery rate (FDR) method for all the statistical tests [33]; an FDR of 10% was carried out.

## Results

Table 1 presents the summary of the correlation between IgG sub-class levels. There were significant positive correlations between all measured IgG subclasses (*P* < 0.0001). In particular, IgG1 and IgG3 levels were highly correlated (r = 0.631). The best correlations were between IgG levels and IgG1 levels on the one hand and IgG levels and IgG3 levels on the other hand (r >0.7).

**Table 1:** Correlation between anti-malarial IgG subclass levels in individuals living in an endemic area in Burkina Faso

	**IgG1**	**IgG2**	**IgG3**	**IgG4**
IgG1	1			
IgG2	0.373 ^a^	1		
IgG3	0.631 ^a^	0.558 ^a^	1	
IgG4	0.467 ^a^	0.326 ^a^	0.46 ^a^	1
IgG	0.71 ^a^	0.537 ^a^	0.787 ^a^	0.507 ^a^

^a^Pearson's correlation coefficient with *P* < 0.0001.

Allele frequencies for all single-nucleotide polymorphisms (SNPs) and previously reported family-based associations with parasitaemia and mild malaria are shown in Table 2 and Additional file 1, respectively. Additional file 1 also shows the family-based associations of SNPs with parasitaemia and mild malaria in individuals (n = 193), for which the level of IgG subclasses has been measured. HbC, *LTA* + 80, *TNF-*1031, *TNF*-238, *TNF*1304, and *NCR3*-412 were associated with parasitaemia or mild malaria in this sub-population ( Additional file 1). Moreover, FcγRIIa H/R131 was associated with mild malaria in individuals, for which the level of IgG2 was higher than the median ( Additional file 1).

**Table 2:** **Family-based association analyses for anti-*Plasmodium falciparum *IgG levels**

**Locus**	**SNP ID number**	**Allele**^**a**^**(AF**^**b**^**)**	**P**^**c**^
*IL12Bpro* (promoter)	rs17860508	TTAGAG/GC (0.3068)	P > 0.05
*IL12B* (3'UTR)	rs3212227	A/C (0.3275)	P > 0.05
*IL4*-590 (promoter)	rs2243250	C/T (0.8017)	P > 0.05
*FCGR2A* H/R131 (exon 4)	rs1801274	C/T (0.5371)	T^d^ (0.0166^f^)
*HbC* (exon1)	rs33930165	A/C (0.1682)	**C**^**d**^**(0.0091**^**e,g**^**)**
*HbS* (exon1)	rs334	A/T (0.0193)	P > 0.05
*LTA* + 80 (intron 1)	rs2239704	C/A (0.3542)	P > 0.05
*TNF-*1031(promoter)	rs1799964	T/C (0.1106)	P > 0.05
*TNF*-863 (promoter)	rs1800630	C/A (0.0858)	A^d^ (0.0440^e^)
*TNF*-857 (promoter)	rs1799724	C/T (0.0228)	**T**^**d**^**(0.0064**^**e,g**^**)**
*TNF*-308 (promoter)	rs1800629	G/A (0.1063)	P > 0.05
*TNF*-238 (promoter)	rs361525	G/A (0.0264)	P > 0.05
*TNF*1304 (intron 3)	rs3093664	A/G (0.075)	**A**^**d**^**(0.0083**^**e,g**^**)**
*NCR3*-412 (promoter)	rs27362191	G/C (0.2267)	P > 0.05
NCR3*3790 (3'UTR)	rs986475	T/C (0.0272)	C^d^ (0.0307^e^)

Abbreviations: HbC, Haemoglobin C; IL4, Interleukin 4; LTA, Lymphotoxin-α; AF, allele frequency; NCR3, Natural cytotoxicity receptor 3; SNP, Single-nucleotide polymorphism; TNF, Tumour necrosis factor; UTR, Untranslated region.

^a^Wild allele / variant allele.

^b^Variant allele frequency that was calculated in the study population.

^c^Chi-square test P-value for IgG levels.

^d^Allele positively associated.

^e^Additive model.

^f^Dominant model.

^g^Significant P value after applying a false discovery rate of 10%.

The family-based association of SNPs with the level of IgG subclasses (IgG1, IgG2, IgG3, and IgG4) and IgG against *P. falciparum* crude extract was further assessed. The additive, the dominant, and the recessive models were used for each SNP. The FBAT analysis gave a number of significant results at the nominal level of 0.05, although there was no association of HbS, *IL12B*pro, and *IL12B* 3’ UTR, *IL4*-590, *NCR3*-412, *NCR3*-3790, *TNF*-238, *TNF*-308, and *TNF-*1031 polymorphisms with the IgG responses (Tables 2 and 3). After correcting for multiple tests, there were only some SNPs that were significantly associated with anti-malarial IgG subclass levels, as shown in Tables 2 and 3. The most significant finding was a positive association between IgG2 levels and FcγRIIa H131 based on a recessive model. Conversely, IgG3 levels were negatively associated with FcγRIIa H131 based on a dominant model. HbC was positively associated with IgG levels based on an additive model. Three *TNF* polymorphisms were associated with IgG responses based on an additive model: *TNF*-863 and *TNF*-857 were associated with IgG3 levels and IgG levels, respectively, whereas *TNF*1304 was associated with IgG3, IgG4, and IgG levels.

**Table 3:** **Family-based association analyses for anti-*Plasmodium falciparum *IgG subclass levels**

	**IgG1**	**IgG2**	**IgG3**	**IgG4**
Locus	P^a^	P^a^	P^a^	P^a^
*IL12Bpro*	P > 0.05	P > 0.05	P > 0.05	P > 0.05
*IL12B3’UTR*	P > 0.05	P > 0.05	P > 0.05	P > 0.05
*IL4*-590	P > 0.05	P > 0.05	C^b^ (0.0472^d^)	P > 0.05
*FCGR2A* H/R131	P > 0.05	**T**^**b**^**(0.0003**^**e,f**^**)**	**C**^**b**^**(0.0010**^**d,f**^**)**	P > 0.05
*HbC*	P > 0.05	C^b^ (0.0139^d^)	P > 0.05	P > 0.05
*HbS*	P > 0.05	P > 0.05	P > 0.05	P > 0.05
*LTA + 80*	A^b^ (0.0305^c^)	A^b^ (0.0505^c^)	A^b^ (0.0214^c^)	A^b^ (0.0139^c^)
*TNF-*1031	P > 0.05	P > 0.05	P > 0.05	P > 0.05
*TNF*-863	A^b^ (0.0379^c^)	P > 0.05	**A**^**b**^**(0.0082**^**c,f**^**)**	P > 0.05
*TNF*-857	P > 0.05	P > 0.05	T^b^ (0.0413^c^)	T^b^ (0.0249^c^)
*TNF*-308	P > 0.05	P > 0.05	P > 0.05	P > 0.05
*TNF*-238	P > 0.05	P > 0.05	P > 0.05	P > 0.05
*TNF*1304	A^b^ (0.0495^c^)	P > 0.05	**A**^**b**^**(0.0099**^**c,f**^**)**	**A**^**b**^**(0.0108**^**c,f**^**)**
*NCR3*-412	P > 0.05	P > 0.05	P > 0.05	P > 0.05
NCR3*3790	P > 0.05	P > 0.05	C^b^ (0.0276^c^)	C^b^ (0.0196^c^)

Abbreviations: HbC, Haemoglobin C; IgG, Immunoglobulin G; IL4, Interleukin 4; LTA, Lymphotoxin-α; NCR3, Natural cytotoxicity receptor 3; *P. falciparum*, *Plasmodium falciparum*; TNF, Tumour necrosis factor.

^a^Chi-square test P-value.

^b^Allele positively associated.

^c^Additive model.

^d^Dominant model.

^e^Recessive model.

^f^Significant P value after applying a false discovery rate of 10%.

## Discussion

The aim of this study was to determine the effect of several candidate gene polymorphisms on the IgG subclass responses against *P. falciparum* extract in a population living in an endemic area in Burkina Faso. Most of the candidates have been associated with parasitaemia, mild or severe malaria [1].

There was positive correlation between all measured antibody levels. Anti-malarial IgG levels were correlated with all anti-malarial IgG subclasses. The best correlations were the correlations of anti-malarial IgG levels with anti-malarial IgG1 and IgG3 levels. A correlation between all anti-*P. falciparum* IgG subclass levels was observed, the best correlation being between IgG1 and IgG3 levels. The results are similar to a study in Thailand, where anti-malarial IgG3 levels were correlated to IgG1 and IgG2 levels [34]. The results are also coherent with a recent study conducted in Sudan, where anti-malarial IgG levels were correlated with each anti-malarial IgG subclass against malarial antigen, and where there was an age-dependent association of protective IgG2 and IgG3 subclasses [12,35]. Cytokines that are effective in most of IgG subclass production could partly explain the correlations between the IgG subclass levels. IL4 induces switching to ϵ, γ1, γ3, γ4 and also enhances the expression of ϵ, γ1, γ3 and γ4 germline transcript, and the secretion of corresponding proteins [36]. *IL4*-590 polymorphism does not explain, nevertheless, variation in the level of IgG subclasses, suggesting that other *IL4* polymorphisms should be investigated. In addition, other cytokines, such as IL2, IL6, or IL10 are also known to enhance the IgG production *in vitro* by B cells activated by polyclonal activators or plasmodial antigens [37,38]. Besides, IL10, which causes the switch towards IgG1 and IgG3, likely explains the high correlation between IgG1 and IgG3 levels [39]. Interestingly, IgG2 and IgG3 levels were also strongly correlated, whereas the correlation between IgG1 and IgG2 levels was low. IFN-gamma seems to inhibit IgG1 production *in vitro*, and to enhance IgG2 production [40]. Although IFN-gamma is unlikely an IgG2 switch factor, T cells producing IFN-gamma, namely the Th1 (T helper 1) lymphocytes, have been shown to produce an IgG2 switch factor acting on CD40-activated naïve B cells [41]. These observations suggest that cytokines produced by Th1 lymphocytes may collectively increase the production of IgG2. In all, the correlations between IgG subclass levels encourage to further assess the production of cytokines in individual living in endemic areas.

The family-based associations of candidate polymorphisms on the IgG subclass levels were further investigated. There were some significant results after applying a multiple test correction. There was no association of IgG subclass levels with *IL4* and *IL12B* polymorphisms, while others reported an association of anti-malarial IgG levels with *IL4*-590 [20]. There was no association between anti-malarial IgG subclass levels and *NCR3* polymorphisms, although those may alter the production of IFN-gamma by NK cells. The association of *LTA* + 80 with all anti-malarial IgG subclass levels was significant at the nominal level of 5%, but was no more significant after applying a multiple test correction; this polymorphism may, nevertheless, require further investigation to confirm or invalidate the trend.

HbS and HbC have been associated with protection against mild and severe malaria, and it has been proposed that these polymorphisms could increase the elimination of the parasite through acquired immune mechanisms [42]. There was no association between HbS and anti-malarial IgG responses, although other studies provided evidence for a positive association [21,22]. These conflicting results may be explained by the low frequency of HbS in the study population. In contrast, HbC that was negatively associated with maximum parasitemia and mild malaria was positively associated with anti-malarial IgG levels in the same population, further suggesting that *HBB* polymorphisms alter the anti-malarial IgG production. This result is consistent with previous reports also providing evidence for an involvement of HbC in the immune response against infected erythrocytes through enhanced IgG production [43,44]. This further supports the hypothesis that the protection against malaria of HbC may be partly mediated by acquired immunity against malaria, more particularly by IgG-mediated effector mechanisms. Interestingly, there was also a trend for the production of anti-malarial IgG2 levels; although the association was not significant after applying a multiple test correction, this trend may require further attention in other populations.

The FcγRIIa H131 allele has been associated with protection from severe malaria in several populations [1]. Furthermore, it has been demonstrated to be the only receptor for IgG2, the high level of which have been associated with protection against *P. falciparum* malaria in a population living in Burkina Faso [14], and in other populations [45,46]. The FcγRIIa H131 allele has been found to be particularly prevalent in the Fulani, who are less affected by clinical malaria than individuals from other ethnic groups; interestingly, the Fulani also showed high levels of anti-malarial IgG2 [23,47]. On this basis, an effect of the FcγRIIa H131 allele on anti-malarial IgG2 levels was anticipated in this study. The data provided evidence of a significant association of FcγRIIa H131 allele with IgG2 levels based on a recessive model. This family-based result is consistent with population-based results previously published [22,23]. Together, these observations indicate that the FcγRIIa H131 allele at the homozygous state increases the production of anti-malarial IgG2. In the same way, there is a trend for anti-malarial IgG levels based on a recessive model, suggesting that the FcγRIIa H131 allele at the homozygous state increases the production of anti-malarial IgG, although the association was not significant after correcting for multiple tests. Thus, the FcγRIIa H131 allele may increase the phagocytosis by monocytes or macrophages, and may favour the antigen presentation by these cells to the T helper lymphocytes; this hypothesis remains to be assessed. Conversely, there was a negative association of the FcγRIIa H131 allele with anti-malarial IgG3 levels based on recessive model, suggesting that the FcγRIIa H131 allele at the homozygous state diminishes the level of anti-malarial IgG3. In the same way, Israelsson *et al.* found that the FcγRIIa R131 allele carriers in the Fulani had higher anti-malarial IgG3 levels than the individuals homozygous for the FcγRIIa H131 allele. One might speculate that more IgG3 will be present in the circulation of individuals with the FcγRIIa R131 allele, because the FcγRIIa H131 allele has a higher binding than the FcγRIIa R131 allele [48]. Nevertheless, the FcγRIIa H131 allele was associated with higher levels of anti-malarial IgG3 in another study in the Fulani [49]. It is likely that other factors affecting the production of IgG subclass antibodies, such as the cytokines or the antigens used, could partly explain the conflicting results [38].

In previous studies, *TNF* polymorphisms have been related to severe malaria. SNPs at position −1031,-857,-308,-238 and −863 in the promoter region of *TNF* gene exhibit differential associations to malaria and TNF production in different populations suggesting that individual TNF responses may be genetically determined [7,8,50,51]. *TNF*1304 within intron 3 was associated with variation in mild malaria and parasitaemia [10], and *TNF*-308A and *TNF*-238A allele have been associated with high anti-*P. falciparum* antibodies [24,25]. In this study, there was no association of IgG subclass levels with *TNF*-308 and *TNF*-238 polymorphisms. *TNF*-863A and *TNF*-857 T were, however, associated with high anti-malarial IgG3 and IgG levels, respectively. Moreover, there was an association of low IgG3, IgG4, and IgG levels with *TNF*1304, which has been associated with mild malaria and parasitaemia in the same population [10]; this further supports the role of *TNF*1304 on malaria phenotypes, although the effect of the polymorphism on the molecular function or the gene expression has not been reported. In contrast, the molecular effect of polymorphisms within the *TNF* promoter has been studied. Other authors reported that the transcription factor OCT-1 binds specifically to the *TNF*-863A and *TNF*-857 T alleles, that the transcriptional promoter activity of a haplotype harbouring *TNF*-863A and *TNF*-857 T was higher than the one of other haplotypes in Japan [50,51]. An explanation for association of *TNF* polymorphism with IgG response could be that *TNF* gene is important in the development of humoral response as an autocrine B-cell growth factor [52].

## Conclusions

Our results indicate that HbC, *TNF*-857T, and *TNF*1304A were associated with high levels of anti-malarial IgG. The FcγRIIa H131 allele was associated with high levels of anti-malarial IgG2 and low levels of anti-malarial IgG3. *TNF*-863A was associated with high levels of anti-malarial IgG3, whereas *TNF*1304 was associated with variation in the levels of anti-malarial IgG3 and IgG4. It should be stressed that all these polymorphisms have been associated with parasitaemia, mild malaria or severe malaria; this suggests that their protective effect may be partly due to their effect on the IgG subclass production.

## Abbreviations

FCGR2A: Fc-gamma Receptor IIA; FDR: False discovery rate; HbC: Haemoglobin C; HbS: Haemoglobin S; IL: Interleukin; IgG: immunoglobulin G; LTA: Lymphotoxin α; NCR3: Natural cytotoxicity receptor 3; P. falciparum: Plasmodium falciparum; TNF: Tumor necrosis factor; UTR: Untranslated region.

## Competing interests

The authors declare that they have no conflicting interests.

## Authors’ contributions

SA calculated the IgG subclass phenotypes, evaluated the correlation between IgG subclass phenotypes, and carried out most of the FBAT analyses. AA and SG participated in the FBAT analyses, and the multiple test correction. FF participated in the design of the study, and revised the results and the manuscript. PR performed the design of the study, supervised the IgG phenotype determination and the statistical analyses, and wrote the manuscript. All authors read and approved the final manuscript.

## Supplementary Material

Additional file 1 Family-based association of polymorphisms with parasitological and clinical phenotypes. Click here for file
